# Application of group-based trajectory models to evaluate the association of fetal growth trajectories and childhood overweight and obesity: A longitudinal study with 2-year follow-up

**DOI:** 10.1371/journal.pone.0330715

**Published:** 2025-09-17

**Authors:** Xiangli Meng, Jingjing Wang, Na Zhang, Xiaofei Li, Qingqing Wu

**Affiliations:** Department of Ultrasound, Beijing Obstetrics and Gynecology Hospital, Capital Medical University, Beijing Maternal and Child Health Care Hospital, Beijing, P.R. China; Kobe University Graduate School of Medicine School of Medicine, JAPAN

## Abstract

**Objective:**

This study investigated whether fetal growth trajectory (FGT) can predict adverse childhood growth outcomes. The correlation between maternal metabolic factors (e.g., body mass index (BMI), diabetes during pregnancy) and FGT was evaluated.

**Method:**

1525 women with singleton pregnancies were included in this study. Fetal growth was assessed at least three times at 20–24 weeks of gestation, 28–32 weeks, and ≥36 weeks. Children’s growth was monitored from birth to 2 years old. A population-based trajectory model was used to analyze the changes in FGT with gestational age and the association between FGT and overweight and obesity in children at 2 years old. Multivariate logistic regression was used to analyze the risk factors affecting fetal growth trajectory. Odds ratio (OR) with its 95% confidence interval (CI) was calculated.

**Results:**

Among the 1525 participants, trajectory analysis identified three FGTs based on fetal estimated fetal weight: an “accelerated growth trajectory” (n = 278, 18.2%), “median growth trajectory” (n = 708, 46.4%), and a “faltering growth trajectory” (n = 539, 35.4%)”. The fetus in accelerating growth trajectory faced fourth the likelihood of being overweight or obesity at 2 years old (RR = 4.22, 95% CI = 2.23, 8.00). Advanced age (OR 1.07, 95%CI 1.0–1.11), high BMI (OR 1.10, 95%CI 1.05–1.14), gestational weight gain (OR 1.05, 95%CI 1.02–1.08)), diabetes during pregnancy (OR 1.74, 95%CI 1.14–2.65)) were risk factors for accelerating growth trajectory.

**Conclusion:**

These findings suggested that accelerating growth trajectory was associated with overweight or obesity in early childhood, and differences in maternal metabolic factors may alter fetal growth trajectory. This study aids in the early identification of infants susceptible to overweight or obesity.

## Introduction

With the recent significant increase and earlier onset of overweight and obesity among children worldwide, the issue of overweight and obese infants and young children has become a global public health challenge. Over the past four decades, global rates of childhood overweight and obesity has soared from 4% to 18%, with the number of obese children increasing more than tenfold [1]. Overweight and obesity affect children’s growth, development, and mental health significantly increasing the risk of chronic diseases such as diabetes and hypertension in adulthood [2]. Notably, the first 1000 days of life (conception through 24 months of age), are widely acknowledged as a life-stage that bridges the fetal period, infancy, and early childhood, during which essential growth and development take place. This period is not only related to the growth and development level during infancy but also closely associated with chronic non-communicable diseases such as obesity, hypertension, and diabetes in adulthood [3]. It is also known as the “window of opportunity” for preventing long term non-infectious illness in later life [4,5]. Consequently, identifying the risk factors and crucial developmental period during early childhood is vital for preventing these adverse growth outcomes.

Fetal growth is a complex and evolving process characterized by cell proliferation, tissue differentiation, and the formation of organs, all of which are critical for establishing a foundation for lifelong health. Fetal adaptive growth at various periods influenced by multiple factors may result in different fetal growth trajectories (FGTs) [6]. Given that organs undergo distinct stages of developmental sensitivity during fetal growth, different FGTs may result in distinct growth patterns, adiposity, visual and neurodevelopmental outcomes in early childhood [7]. Longitudinal ultrasound measurements of fetal growth can provide valuable information on the FGTs and help find connections between these patterns and later childhood growth. Currently, few studies have explored this correlation [8]. Additionally, maternal metabolic factors such as prepregnancy BMI and diabetes during pregnancy play a significant role in influencing fetal growth patterns [9,10]. Neverthless, few studies have investigated whether the relationship between fetal growth and adverse child growth outcomes varies depending on maternal metabolic conditions.

Estimated fetal weight (EFW) is one of the most frequently utilized indicators for evaluation of fetus’s overall growth status. In this study, group-based trajectory modeling (GBTM) was used to identify fetuses with similar growth trajectories. Therefore, this study aimed to identify FGTs based on longitudinal EFW to identify high-risk trajectories associated with overweight and obesity at two years old and to screen clinical features related to high-risk FGTs.

## Methods

### Study population

**Fig 1:**
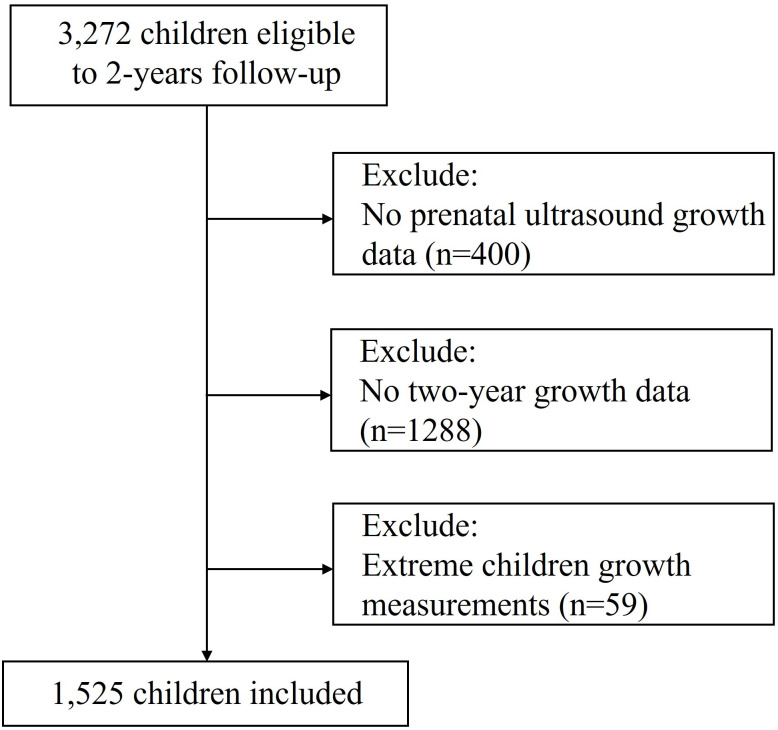
Flowchart of participant inclusion.

The study population of 3272 women was recruited from January 2019 to December 2019 at Beijing Obstetrics and Gynecology Hospital Capital Medical University (BJOGH). The fetuses were tracked throughout pregnancy until birth, and their children’s health and growth were monitored up to 2 years old, with corrected age calculations applied for preterm infants. The inclusion criteria for participants were as follows: (1) Singleton pregnancies with accurate gestational age (estimated based on a reliable last menstrual period (LMP), defined as a discrepancy ≤ 7 days between gestational age calculated using LMP and ultrasound, (2) underwent at least 3 ultrasound examinations during routine pregnancy scans (20−24, 28−32, 36–41 weeks), (3) delivered a newborn without any congenital malformations, and (4) intended to deliver at BJOGH and stay in Beijing for a minimum of 2 years after delivery. The exclusion criteria for participants were: (1) prenatal ultrasound growth parameters were incomplete, (2) children lacking 2-year growth data, and (3) extreme children growth measurements following WHO growth standard recommendations [11]. Ultimately, we enrolled 1525 children for final analysis. The flow of participants is illustrated in Fig 1. This study was approved by the Beijing Obstetrics and Gynecology Hospital, Capital Medical University Ethics Committee (NO.2018-KY-003–03). All participants were provided with information regarding the study and gave their written informed consent prior to participation. This study was conducted in compliance with the Declaration of Helsinki and all applicable ethical guidelines.

### Fetal ultrasonography

Fetal biometry measurements including head circumference, abdominal circumference, and femur length were obtained from the ultrasound records documented in the electronic health system. Fetal biometry parameters were measured following ISUOG guidelines [12]. Additionally, EFW was calculated using the National Institute of Child Health and Human Development (NICHD)-Asian formula [13]. Moreover, EFW percentiles were calculated using previously reported method for singleton fetal growth and converted into z scores using a free online calculator [13]. In this study, all ultrasound measurements were taken to the millimeter. Moreover, the sonographers responsible for these measurements were certified in operating the relevant medical devices and possessed a minimum of three years of experience in fetal ultrasonography. To maintain acceptable levels of intraobserver and interobserver variability, the quality control team at the participating hospital conducted monthly reviews by randomly selecting 10% of the scans for blinded evaluation.

### Evaluation of child growth outcomes

Anthropometric weight and length measurements were serially collected during the first 2 years through routine physical examination using standard anthropometric methods [14]. The child’s weight and length were measured twice each visit, and the average was used as the final measure. Only data from children with anthropometric measurements for both weight and length at the following age points were used in this analysis: 6 months, 12 months, 18 months, and 24 months. We calculated continuous outcomes (length-for-age Z-score, LAZ; weight-for-age Z-score, WAZ; and weight-for-length Z-score, WLZ) using WHO Child Growth Standards [15]. Children with WLZ greater than 2 and 3 were defined as overweight and obese, respectively [16].

### Covariates and other information

Data on maternal age at conception, pre-pregnancy weight, height, parity (nulliparous or multiparous), and LMP were gathered through self-reported questionnaires during recruitment (around 12 weeks gestation). Pre-pregnancy BMI was calculated by dividing weight in kilograms by the square of the height in meters before conception. Details on maternal pregnancy complications, such as diabetes (including pregestational and gestational diabetes) and hypertensive disorders during pregnancy (HDP), as well as child’s sex, date of birth, birth weight, and birth length were extracted from medical records. Gestational diabetes was diagnosed based on the criteria established by the International Association of Diabetes and Pregnancy Study Group [17].

### Statistical analysis

To establish the growth EFW growth trajectories, we developed models for EFW z scores using GBTM. As such, we used the “PROC TRAJ” SAS package to conduct GBTM [18]. A predetermined maximum of six trajectory groups was established. We fitted the models from one group trajectory to six group trajectories, with gestational age serving as a timescale. A z-score of 0 at 10 weeks gestation was assigned to each fetus to emphasize growth trajectories in relation to the initial ultrasound measurement, ensuring grouping was based solely on size. In order to determine the model featuring an optimal number of distinct EFW trajectories, we initially crafted longitudinal EFW z score trajectories by employing a polynomial model (extending up to cubic models) for each EFW z score, with gestational age serving as the independent variable. Subsequently, we evaluated the Bayesian information criteria (BIC) value to identify the model that provided the best fit. The final trajectory model was designed to meet the following diagnostic criteria: (1) the average posterior probability of assignment in all groups ≥ 70%; (2) Posterior probability of group membership greater than ≥ 5%; and (3) the odds of correct classification in all groups > 5.0; (4) Relative entropy greater than 0.8 [19]. We then used multinomial logistic regression to evaluate predictors (dependent) associated with each trajectory group (independent). Finally, we estimated the risk ratios (RRs) and associated 95% confidence intervals (95% CIs) for FGTs and childhood overweight and obesity. Predictors would be non-significantly or significantly associated with a particular trajectory group (significance level was set at p = 0.05).

Continuous variables with normal distribution are presented as mean ± standard deviation and were compared using the t-test. Continuous variables with skewed distributions are presented as medians with interquartile ranges and compared with the Mann–Whitney U or Kruskal–Wallis H tests. Categorical variables are presented as numbers with percentages and compared with the chi-square test. All statistical analyses were performed using SAS (version 9.4; SAS Institute Inc, Cary, NC, USA).

## Results

### Baseline characteristics

From January 2019 to December 2019, we screened 3272 pregnant women for eligibility, and 1525 were enrolled in the trial (Fig 1). The participant non-attendance at the 2-year follow-up mainly due to the reduced motivation for follow-up at the cohort clinic and the lockdowns during the COVID-19 pandemic or due to illness. The mean age of the 1525 participants was 31.0 years. Of the sample, the majority of whom were nulliparous: 8.2% were HDP, and 11.4% had diabetes during pregnancy. The prevalence of childhood overweight and obesity was 2.9%. The baseline characteristics are presented in Table 1.

**Table 1:** Baseline characteristics of study cohort according to fetal growth trajectories.

Variables	All N = 1525	Faltering growth N = 278	Median growth N = 708	Accelerating growth N = 539	*P*
**Maternal characteristics**					
Age (years)	31.0(29.0,34.0)	31.0(29.0,34.0)	31.0(29.0,33.0)	32.0(30.0,35.0)	<0.001
Height (m)	1.63(1.60,1.67)	1.61(1.58,1.64)	1.63(1.60,1.67)	1.65(1.61,1.68)	<0.001
Weight (Kg)	57.0(52.0,65.0)	53.0(48.0,58.0)	55.5(51.0,63.0)	62.0(55.0,69.0)	<0.001
Prepregnancy BMI (Kg/m^2)^	21.5(19.5,24.0)	20.4(18.5,22.3)	21.1(19.3,23.5)	22.7(20.6,24.9)	<0.001
<18.5	217(14.2)	68(24.5)	112(15.8)	37(6.9)	
18.5-24.9(%)	1038(68.1)	188(67.6)	482(68.1)	368(68.3)	
25-29.9	220(14.4)	20(7.2)	91(12.9)	109(20.2)	
≥30	50(3.3)	2(0.7)	23(3.2)	25(4.6)	
Weight gain during pregnancy (Kg)	14.0(11.0,17.0)	13.5(10.0,17.0)	14.0(11.5,17.0)	14.5(11.5,18.0)	<0.001
Nulliparous n(%)	1116(73.18)	235(84.53)	540(76.27)	341(63.27)	<0.001
Hypertensive disorder in pregnancy n(%)	125(8.20)	50(17.99)	33(4.66)	42(7.79)	<0.001
Diabetes during pregnancy n(%)	174(11.41)	30(10.79)	54(7.63)	90(16.70)	<0.001
**Child characteristics**					
Gestational week at delivery, (weeks)	39.0(38.0,40.0)	39.0(38.0,40.0)	39.0(39.0,40.0)	39.0(38.0,40.0)	<0.001
Birth weight (Kg)	3.46(2.86,4.14)	2.62(2.35,2.79)	3.30(2.92,3.76)	4.15(3.79,4.33)	<0.001
Birth length (cm)	50.0(49.0,52.0)	48.0(46.0,49.0)	50.0(49.0,51.0)	52.0(51.0,53.0)	<0.001
Birth weight: placental weight ratio	5.41(4.81,6.13)	5.00(4.47,5.54)	5.52(4.95,6.10)	5.56(4.81,6.54)	<0.001
Child’s sex, Male n(%)	726(47.61)	106(38.13)	302(42.66)	318(59.00)	<0.001
Overweight or obesity at 2 years of age	44(2.89)	2(0.72)	11(1.55)	31(5.75)	<0.001
Overweigh at 2 years of age (%)	38(84.1)	2(100)	10(90.9)	26(83.9)	<0.001
Obesity at 2 years of age (%)	6(15.9)	0(0)	1(9.1)	5(16.1)	0.042

Data are n (%), or median (IQR). P-values were obtained by the Mann-Whitney U test, or Chi-squared test wherever appropriate comparing among groups; BMI, body mass index. BMI, body mass index.

### Fetal and early childhood growth trajectories

**Fig 2:**
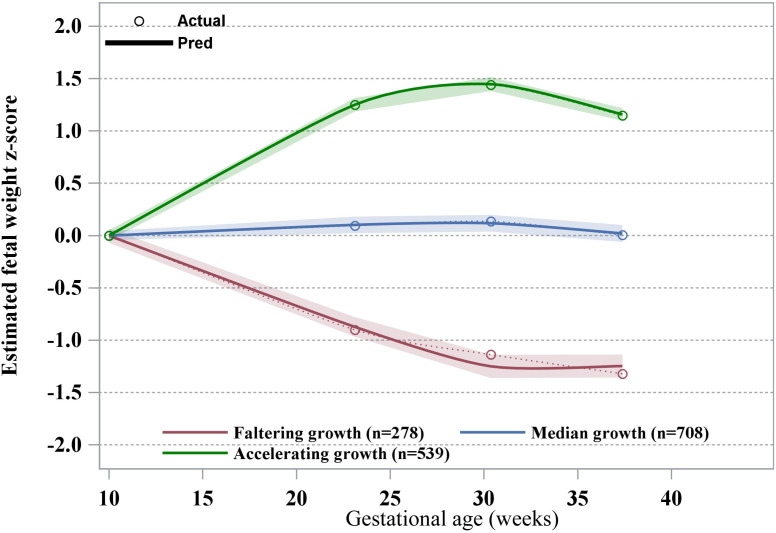
Fetal growth trajectories.

When the number of latent class groups (the number of subgroups) increased from 1 to 6, BIC gradually decreased. However, when the number of subgroups was 5 and 6, the sample size in one of the subgroups was too low (less than 5% of the total sample size). According to the principle of model parsimony, we identified the GBTM model with three trajectories as the optimal model. Moreover, the average posterior probability of the three groups were 90.79, 89.84 and 93.53, respectively, which were all greater than the empirical standard of 0.70, indicating that the model fitting was better. The summary of model fitting information was summarized in supplementary S1 Table. Fig 2 showed three longitudinal patterns of EFW growth: “faltering growth” (n = 278, 18.2%), “median growth” (n = 708, 46.4%), and “accelerating growth” (n = 539, 35.3%). The reference group was the median growth, aligning closely with the 50th percentile. The faltering growth exhibited a decline in EFW from early pregnancy until 30 weeks gestation, after which the trajectory remained slightly below –1 SD from the mean. In contrast, the accelerating growth exhibited rapid growth, reaching +1.5 SD from the mean by 30 weeks of gestation, mirroring the pattern of faltering growth but in the opposite direction.

**Fig 3:**
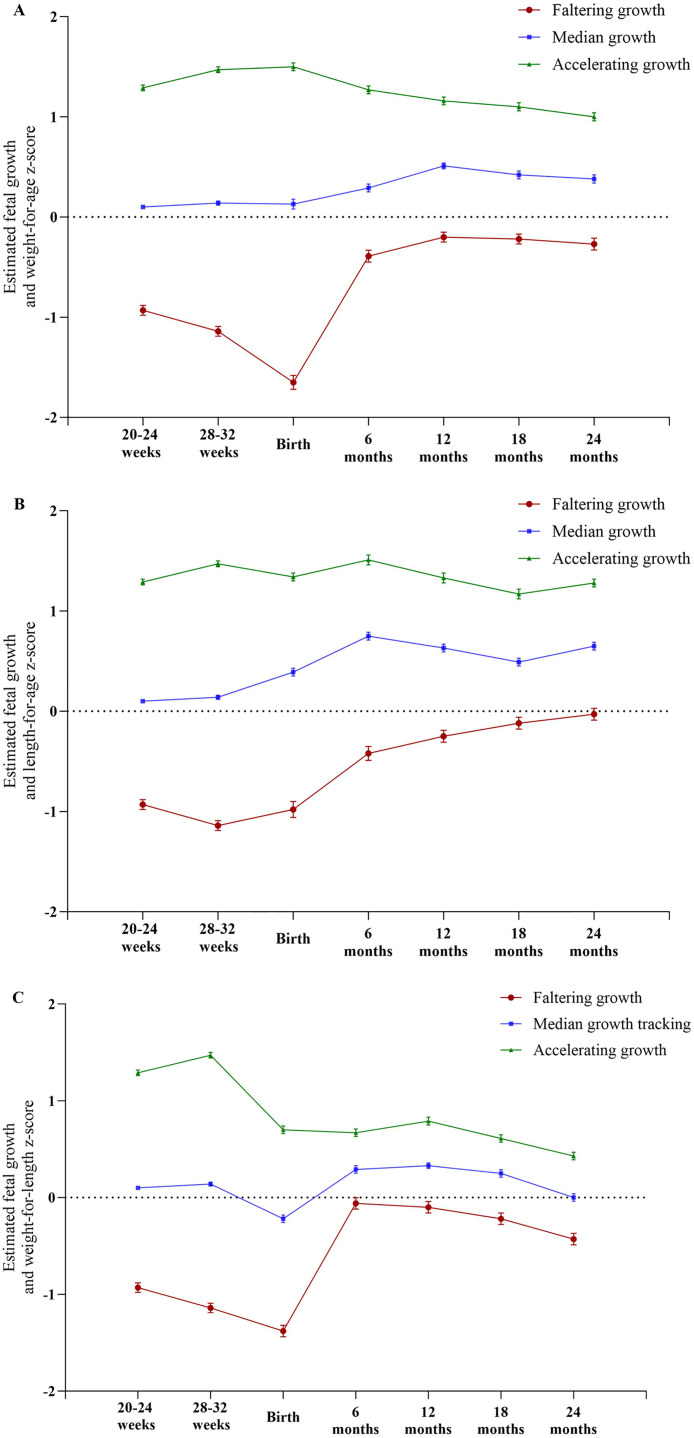
Growth z-scores for estimated fetal weight during pregnancy, and weight (A), length (B), and weight-for-length (C) at birth, 6 months, 12 months, 18 months, and 24 months.

For each FGT, the following measures were evaluated: (1) EFW measured between 20–24 weeks and 28–32 weeks gestation; (2) birthweight and birth length adjusted for gestational age and sex expressed as z scores; and (3) LAZ, WAZ and WLZ at age 6 months, 12 months, 18 months and 24 months, all expressed as z-scores based on the WHO Child Growth Standards ^(10)^. From the second trimester of pregnancy until 2 years old, the faltering and accelerating growth trajectories diverged from the median growth trajectory (Fig 3A, B). A comparable trend was observed for WLZ at 1 and 2 years old as an indicator of early childhood overweight and obesity (Fig 3C); however, a slight downward shift across the three FGTs suggested a reduced rate of weight gain relative to length, particularly in the accelerating growth trajectory.

### Trajectory sub-population characteristics and child growth outcome

Table 1 presents the baseline characteristics of participants within each FGT. Participants in the “accelerating growth” trajectory group were more likely to be older, had higher pre-pregnancy BMI, and had a high proportion of diabetes during pregnancy and high birth weight compared with those in the “median growth” and “faltering growth” group). Regarding anthropometry measurements, an accelerating growth trajectory was significantly linked to higher WLZ scores at 2 years old. Furthermore, it increased the likelihood of being overweight or obese (RR = 4.22, 95%CI = 2.23–8.00), as well as overweight (RR = 4.36, 95% CI = 2.17, 8.75) and obese (RR = 9.15, 95% CI 1.07, 78.09) by the same age.

### Identification of risk factors associated with accelerating growth trajectory

**Fig 4:**
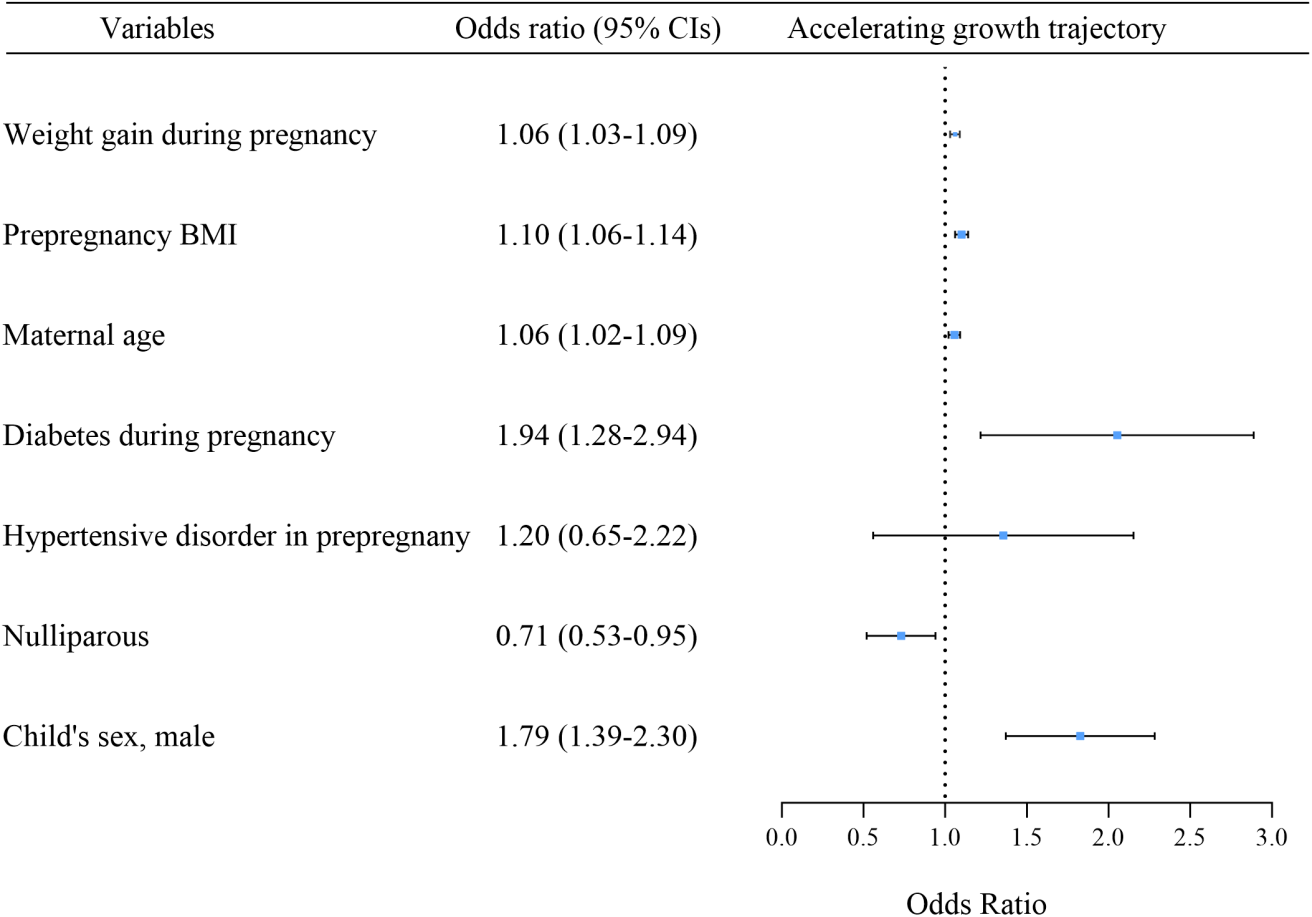
Associations between variables and accelerating growth trajectory. BMI, body mass index.

The analysis using multinomial logistic regression revealed that fetal sex (male), maternal BMI, diabetes during pregnancy, maternal age, and maternal weight gain during pregnancy were risk factors for accelerating growth trajectory. The OR (95% CI) values were as follows: fetal sex (male) 1.79 (1.39–2.30), pre pregnancy BMI 1.10 (1.06–1.14), diabetes during pregnancy 1.94 (1.28–2.94), maternal age 1.06 (1.02–1.09), and weight gain during pregnancy 1.06 (1.03–1.09). Additionally, primiparity could reduce the risk of accelerating growth trajectory, with an OR (95% CI) value of 0.71 (0.53–0.95) (Fig 4).

## Discussion

This study identified that accelerating growth trajectory increase the risk of being overweight or obesity at 2 years old by fourfold compared to children who present a nonaccelerating growth trajectory. Maternal pre-pregnancy BMI, age, weight gain during pregnancy, and diabetes during pregnancy were risk factors for accelerating growth trajectory, suggesting that the association between fetal growth and adverse child growth outcomes may vary depending on maternal metabolic status.

Our findings indicate that accelerating growth trajectory, characterized by increased fetal size during pregnancy, is linked to a higher risk of early childhood overweight or obesity, consistent with previous studies. Moreover, a higher birth weight has been linked to an elevated likelihood of childhood overweight [20]. While many studies used birthweight as a proxy for fetal growth, we hypothesize that this a static measure that fails to capture growth patterns prior to birth. Based on the INTERGROWTH-21st Project established international standards for fetal head and abdominal circumferences [21], one study demonstrated that accelerating fetal abdominal circumference growth trajectories were linked with obesity by age two [7]. Similarly, a Chinese cohort study found that a larger EFW throughout pregnancy correlated with a higher risk of being overweight and obese at 3 years old [8]. Furthermore, previous studies have noted that reduced triglyceride concentrations in LGA fetuses may indicate advanced prenatal fat tissue development and overnutrition during pregnancy [22,23]. Thus, alterations in metabolites related to lipid metabolism pathways in umbilical cord blood, associated to accelerated FGTs, may predispose children to obesity.

Importantly, this study explored risk factors linked to accelerating growth trajectory, some of which are adjustable and could act as potential targets for intervention. These risk factors included maternal age at conception, pre-pregnancy BMI, weight gain during pregnancy, and blood glucose levels during pregnancy. Studies have shown that high pre-pregnancy BMI and excessive gestational weight gain were significant risk factors for overweight and obesity in children [24,25]. Diabetes during pregnancy, as the most common complication of pregnancy, often leads to excessive growth of offspring [26]. These factors may play a role in a variety of mechanisms, such as changing maternal and fetal metabolic pathways, affecting the levels of insulin and other growth factors, and the fetal epigenome [27–29]. These factors may also contribute to accelerated growth from the fetal stage through early childhood and maintain an above-median growth trajectory, increasing the risk of being overweight and obese by age two. As a result, it is recommended that women managed their weight before pregnancy, weight gain, and blood glucose levels during pregnancy, and pregnant women with overweight and obesity or diabetes should pay close attention to fetal growth trajectory to intervene in early life to prevent childhood obesity.

The strength of this study lies in the use of advanced statistical models (namely, GBTM) to analyze FGTs. Notable, this approach enabled the identification of distinct groups of individuals with similar growth patterns and levels over time. In contrast, linear mixed models primarily focus on average population trajectories. Nevertheless, there are several limitations. First, being a retrospective study, there is a possibility that missing data and selection bias could influence the findings. Secondly, the study sample was drawn from a single center, which may restrict its generalizability. However, Beijing, as the capital of China, has a relatively diverse population, including people from all over the country. This may somewhat mitigate some of the limitations of single-center studies. Third, this study did not account for fetal growth during the first trimester, possibly representing another crucial phase of fetal development. Fourth, the potential for residual confounding, such as infant feeding practices, the time of complementary feeding, and/or parental BMI, cannot be excluded, which may introduce bias into the results, and further prospective studies will combine with these parameters. We also lacked lifestyle information on excessive physical activity, stress, and malnutrition, which may lead to a deviation in our estimation of the relationship between fetal growth trajectory and the overweight and obesity risk of early childhood. Thus, this should be examined in future studies. Finally, the metabolite differences between different FGTs were not included in the analysis of this study, so further studies are needed.

## Conclusion

The risk of obesity or overweight at 2 years old in accelerating growth trajectory was fourfold higher than that of nonaccelerating growth tracking. Maternal age, pre-pregnancy BMI, gestational weight gain during pregnancy, child’s male sex, and diabetes during pregnancy were independent risk factors for accelerating growth trajectory. These may affect fetal growth and prevent adverse outcomes in early childhood. Future studies are needed to determine whether the growth outcomes of follow-up FGTs persist into adolescence and adulthood.

## Supporting information

S1 Table Parameters for estimated fetal weight growth trajectory group (N = 1525). (DOCX)
