# Supplementary material for: Application of group-based trajectory models to evaluate the association of fetal growth trajectories and childhood overweight and obesity: A longitudinal study with 2-year follow-up
Source: PLoS One. 2025 Sep 17;20(9):e0330715. doi: 10.1371/journal.pone.0330715 (PMC12443285; doi:10.1371/journal.pone.0330715)
Supplement: S1 Table — (DOCX) [file pone.0330715.s001.docx]

**S1 Table Parameters for estimated fetal weight growth trajectory group (N=1525).**

| Group | Avepp(%) | OCC | P_j_(%) | π_j_(%) | BIC | △BIC | E_k_ |
| --- | --- | --- | --- | --- | --- | --- | --- |
| 1 | 100.00 |  | 100.00 | 100.00 | -8378.27 |  | 0.000 |
| 2 | 94.51-95.67 | 20.3-18.7 | 46.03-53.97 | 45.84-54.16 | -7176.12 | 1202.15 | 0.831 |
| 3 | 90.79-89.84-93.53 | 42.8-10.6-26.0 | 18.23-46.30-35.48 | 18.71-45.56-35.73 | -6800.03 | 376.09 | 0.812 |
| 4 | 88.20-89.85-88.48-91.78 | 11.5-17.0-32.6-143.0 | 39.15-35.02-18.89-6.95 | 39.40-34.27-19.08-7.25 | -6622.19 | 177.84 | 0.812 |
| 5 | 86.99-88.39-83.70-86.85-88.89 | 84.4-16.3-10.2-22.4-162.8 | 7.02-31.61-34.10-22.62-4.66 | 7.34-31.82-33.42-22.73-4.68 | -6547.55 | 74.64 | 0.801 |
| 6 | 89.86-83.77-88.35-77.78-75.91-89.46 | 117.0-10.6-17.3-18.2-33.1-164.5 | 6.43-33.90-30.62-16.00-8.07-4.98 | 7.04-32.71-30.50-16.14-8.70-4.90 | -6511.73 | 35.82 | 0.786 |

Good model fit indicated by (1)Avepp(Average posterior probability)greater than 0.7 for each group,(2)Pj(Posterior probability of group membership) greater than 5%,(3)Close correspondence Pj and πj(Probability of group membership),(4) BIC(Bayesian information criterion) close to 0,(5)The large △BIC(BIC difference between complex and simple models) the more complex models are accepted,(6)OCC(Odds of Correct Classification)greater than 5,(7)Ek(Relative entropy) greater than 0.8
